# Exploration of Friendship Experiences, before and after Illness Onset in Females with Anorexia Nervosa: A Qualitative Study

**DOI:** 10.1371/journal.pone.0163528

**Published:** 2016-09-27

**Authors:** Heather Westwood, Vanessa Lawrence, Caroline Fleming, Kate Tchanturia

**Affiliations:** 1 Psychological Medicine, Institute of Psychiatry, Psychology and Neuroscience, King’s College London, London, United Kingdom; 2 Health Services and Population Research, Institute of Psychiatry, Psychology and Neuroscience, King’s College London, London, United Kingdom; 3 South London and Maudsley NHS Foundation Trust, London, United Kingdom; 4 Ilia State University, Tbilisi, Georgia; Universite de Bretagne Occidentale, FRANCE

## Abstract

**Background:**

Difficulties with social relationships have been implicated in both the development and maintenance of Anorexia Nervosa (AN) but the friendship experiences of individuals with AN have not been explored in depth.

**Method:**

Ten adults with AN took part in a semi-structured interview about their friendship experiences both before and since the onset of their illness.

**Results:**

Five principle themes were identified through thematic analysis: Social Concern; Impact of AN; Social Connectedness; Inflexibility and Preferred Social Activity. Difficulties with friendship were present prior to the onset of AN in all cases, with participants experiencing anxiety in relation to various aspects of their friendships. Participants described mixed experiences of how their AN has affected their friendships but most participants described having less contact with their friends since becoming unwell.

**Conclusion:**

This research highlights the role that social difficulties may play in the development of AN, whilst also emphasising the importance of addressing problems with friendship in the course of inpatient treatment.

## Introduction

Anorexia Nervosa (AN) is a severe eating disorder (ED), characterised by restricted energy intake relative to requirements; undue influence of weight and shape on self-evaluation and fear of gaining weight despite being significantly underweight [1]. Along with the serious physical complications associated with the disorder, several psychological symptoms contribute to, and maintain AN [2]. One such factor is difficulty with social interaction [3]. Treasure and Schmidt [2] describe social avoidance as a core feature in people with AN, with social difficulties often predating the onset of the illness.

Young people who develop EDs have been found to engage in more solitary activities than healthy controls (HC) and are likely to report few or no childhood friends [4], with this behaviour continuing into adulthood [5]. Girls who believe that being thin will improve their friendships are more likely to be concerned about their weight [6], highlighting the socio-cultural risk factors of disordered eating, which may lead to clinical EDs. Bullying is associated with an increased risk of developing AN [7] and peer environment may also impact on body image concern and dieting behaviour; in early adolescence, girls share similarities in dietary restraint and extreme weight loss behaviour with their friends [8]. This highlights the role that friendship experiences may play in the development of an ED but to date, research has not focused on the lived-experience of individuals with AN in terms of their friendships.

Despite the family functioning of patients with EDs being widely explored [9], a lack of studies have examined wider social networks in relation to the development or maintenance of AN [10]. Both peer groups and family are associated with body-image concern and body dissatisfaction. Appearance comparison has also been found to mediate the relationship between peer influences and body image, eating disturbance and self-esteem [11]. Reduced social support from families along with low self-esteem and body image concern constitute a risk profile which can be causally linked to the development of EDs [12] but it is unclear whether individuals with AN perceive wider difficulties within their social networks.

During the acute stage of the illness, patients with EDs have been shown to have smaller social networks than their unaffected peers [13] and report poorer social functioning than non ED controls [3,14]. Adolescents with an eating disorder engage in more body-related social comparison when compared with those with depressive disorder or HCs and this social comparison is directly associated with the severity of eating disorder symptoms even when low self-esteem and depressive symptoms are accounted for [15].

The lived experiences of individuals with AN regarding their friendship experiences is lacking within the literature but an individual who has now recovered from AN summarised her experience in one word–“isolation” [16]. McKnight describes being left behind by her friends and people pretending the AN is not there, while also highlighting the invaluable support of family and friends during the process of recovery. In a qualitative examination of the friendship experiences of females with AN and suspected Autism Spectrum Disorder (ASD), themes arose around: limited social network; difficulty in understanding friendship; lack of communication with friends and focus of attention away from the self [17]. These themes reflect what is reported in the ASD literature but as this study focused on participants suspected to have both AN and ASD, it is not known whether these friendship difficulties are experienced more broadly in AN.

For individuals with severe AN, issues regarding isolation and poor social support may be particularly poignant. Gorse et al. [18] identify isolation as being a reason cited by individuals with AN for seeking hospital treatment, and inpatient care may become iatrogenically reinforcing by temporarily reducing isolation [19]. Individuals with severe and enduring AN report poor quality of life, particularly in the domains of interpersonal avoidance, with the illness having a negative impact on social life [20]. In addition, individuals with AN report finding it difficult to be with people socially despite citing the importance of life outside of work or study as a motivator to recover [21,22], thus an awareness of the social difficulties of individuals with AN may have important implications for recovery and transition from inpatient to community treatment. A recent qualitative study exploring the social functioning of adolescents admitted for inpatient treatment for EDs highlighted development and/or maintenance of social network and interpersonal skills as a key requirement for service provision [23]. This research also highlighted that adolescents with EDs report social difficulties which appear to persist over and above those typically experienced in individuals without EDs, research examining these difficulties in adults would be beneficial to extend this line of research.

Despite the growing evidence base describing the social and emotion difficulties of people with EDs, few interventions specifically target these issues. Davies [24] describes a group-work approach to addressing the friendship issues of inpatient adolescents with EDs, but this work is only described within a pilot programme. Sharpe et al. [25] highlights the need for research which focuses on the social experiences that contribute to the development of eating pathology so that specific prevention programmes, which target peer-related risk factors, may be developed. While specific interventions which target social difficulties may be beneficial, particularly for individuals with severe AN [20], it is important to understand the friendship experiences of individuals with the disorder from their own perspective so that such interventions can be tailored to meet the specific needs of this patient group.

### Aim

The aim of the current study was to qualitatively explore the friendship experiences of individuals hosptialised for AN, both before the onset of their disorder and during the time they have been unwell.

## Materials and Methods

### Design

Semi-structured interviews were used to collect information about individual’s friendship experiences. The thematic analysis method, described by Braun and Clarke [26], was adopted and a data-driven, inductive approach was taken as the friendship experiences of individuals with AN has been relatively unexplored within the literature.

### Participant recruitment

Participants were recruited from a specialist inpatient ED unit. This setting was chosen to reflect the literature on social difficulties in AN, which suggest that individuals receiving hospitalised treatment may experience particularly high levels of social isolation [18]. It was also felt that being in hospital, removed from social contact may allow participants to reflect more openly on their experiences.

Inclusion criteria were kept as broad as possible to reflect the heterogeneous nature of AN and consisted of a primary diagnosis of AN and a good level of spoken English. Purposive sampling was used to obtain a range of ages, illness severity and duration in an attempt to capture the experiences of a variety of patients. Patients on the inpatient ward were informed about the study by HW and of the patients approached, only one declined to participate, stating that she did not want to talk about friendship because it was an upsetting topic for her.

### Data collection

Ethical approval for the study was obtained from an NHS Health Research Authority Ethics Committee (14/LO/2131). Ten semi-structured interviews were conducted on a specialist inpatient ED unit. Written informed consent, including consent to audio-record the interviews was obtained prior to any research procedures.

### Procedure

All interviews were conducted by HW and took place in private on the inpatient ward. Each participant was informed that the purpose of the interview was to explore friendship experiences. Interviews began with the same open question: “Can you tell me about your friendship experiences when you were younger, before you became unwell with Anorexia?” This questions was used in an attempt to elicit responses related to childhood friendship experiences, rather than purely focusing on social life since the development of the disorder. An interview schedule was used to ensure various aspects of friendship were covered including: making friends; friendship activity; worries or difficulties and the impact of AN on friendship. The questions were semi-structured, building on the responses of each participant and efforts were made to follow the participant’s priorities and concerns. The schedule was revised iteratively to best capture areas of particular importance.

### Data analysis

**Fig 1:**
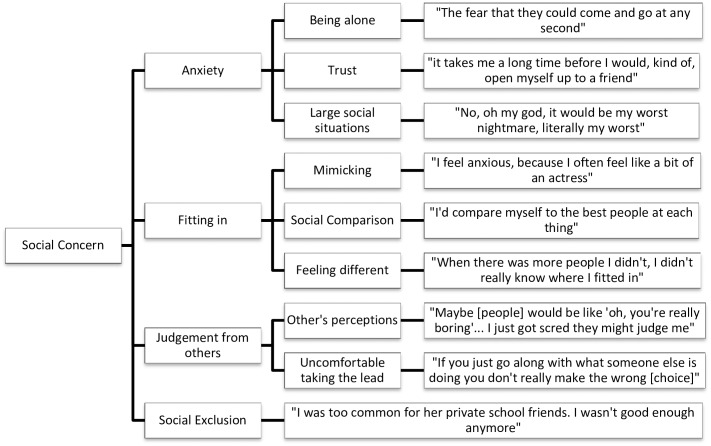
Social Concern thematic map.

**Fig 2:**
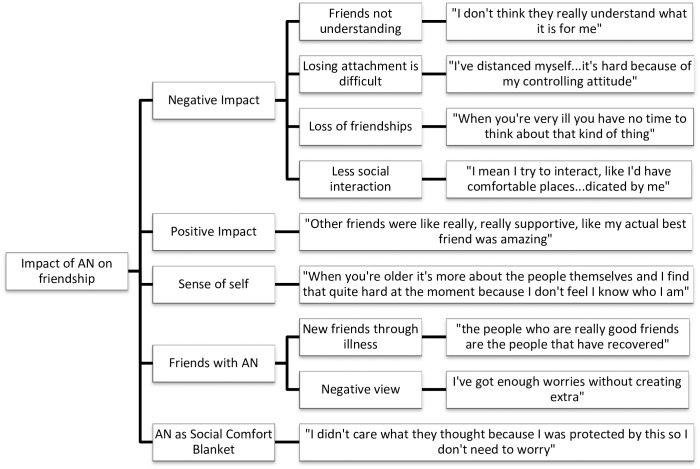
Impact of AN on Friendship thematic map.

**Fig 3:**
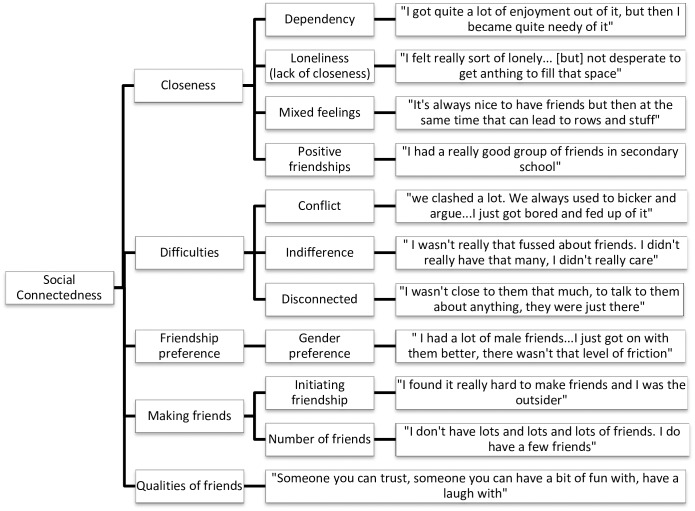
Social Connectedness thematic map.

**Fig 4:**
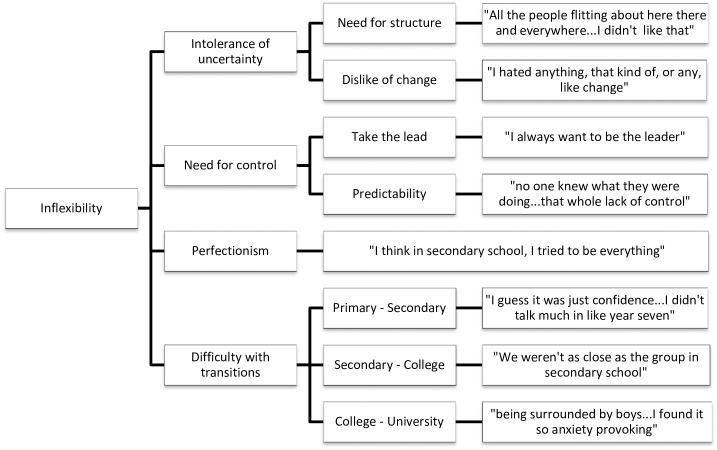
Inflexibility thematic map.

**Fig 5:**
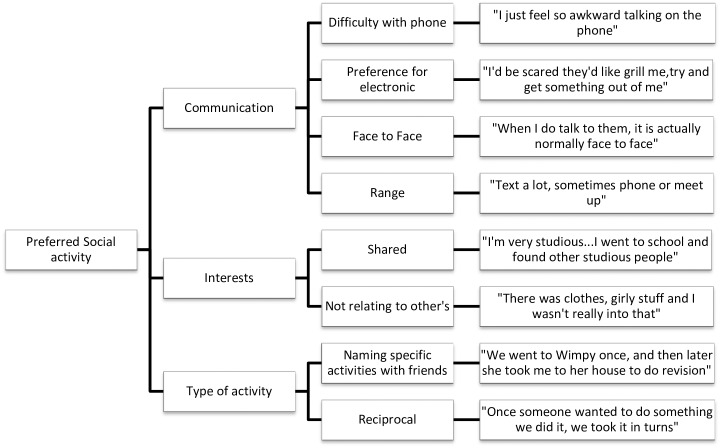
Preferred Social Activity thematic map.

Data analysis was undertaken concurrently with data collection using NVivo QSR International qualitative analysis software, version 10. Interviews were transcribed verbatim by either HW or CF. HW and CF read the transcripts several times to become immersed in the data. Initial coding was done by HW and CF using sentence-by-sentence coding, before HW grouped codes into overarching themes and then divided them into meaningful subthemes. The dataset was reviewed to check how well the themes fit with the original data. Thematic maps were produced to show the links between the overarching themes and subthemes (Figs 1–5).

## Results

### Participants

Participant information is displayed in Table 1; all names have been changed to maintain confidentiality. All participants were female with a primary diagnosis of AN [1]; one participant had a co-morbid anxiety disorder; one had diagnoses of obsessive compulsive disorder and specific phobia and a third had suspected ASD. Nine of the participants were Caucasian and one was Black African. All participants spoke English as their first language.

**Table 1:** Demographic information.

Pseudonym	Age	AN illness duration (years)	Interview length
Nina	20	1	12:11
Darcy	22	9	14:23
Tanya	21	<1	17:17
Sam	42	27	12:14
Emma	19	3	10:52
Tess	30	14	11:47
Olivia	20	4	16:44
Teegan	18	1	13:27
Freya	21	<1	12:31
Danielle	20	2	16:08

### Themes

Five overarching themes were identified through analysis, with each theme being divided into subthemes to fully capture the data. The themes are presented in order of perceived importance and clinical implication. Frequencies of key themes are given to help illustrate patterns within the data and support transparency of data analysis.

#### Social concern

All participants spoke about concerns they currently have or have previously had in relation to friendship, which was split into the subthemes of anxiety, fitting in, judgement and social exclusion. Participants all expressed anxiety about some aspect of their friendship: “well I withdrew from school, it was actually because I ended up, just having almost daily panic attacks; I just found the whole thing completely overwhelming” (Darcy). Others mentioned feeling anxious in social situations: “I only used to mingle if I’d had quite a lot to drink, if not, I couldn’t” (Teegan).

Participants described experiencing it hard to fit in with others or feeling as if they were different. Sam spoke extensively of trying to copy others: “I was trying to act out what I saw on television”. Others spoke about not fitting in with their peers at school: “When there was more people, I didn’t really know where I fitted in” (Darcy). Social comparison was raised in the interviews, particularly in relation to other’s popularity or appearance: “I think I’ve always battled with confidence in the way that I looked…it was that side of me that was quite competitive” (Tanya).

The sub-theme of judgement was coined to describe numerous references to worrying about being judged by others; this was apparent in most of the interviews. Four participants were worried about people thinking badly of them: “You have to dress up and what do you wear? People are dancing and you don’t know, how do you dance?” (Olivia). Other participants spoke about not wanting to take the lead in social situations out of concern that people would judge their decisions negatively. Social exclusion in some form was mentioned by five participants. This ranged from being bullied: “I was excluded from social groups” (Tess) to feeling betrayed.

#### Impact of anorexia nervosa

All but one participant spoke about the negative impact that AN has had on either making or maintaining friendships. Negative affects included friends not understanding the ED: “when you just read like, a magazine article about someone who lost five stone and so I don’t know if they actually know…what it is for me [AN]” (Olivia). Other participants spoke about losing friends as a result of becoming ill: “I didn’t realise what I was losing until I came to hospital” (Tanya). Participants spoke about still having their friends, but having less interaction with them since being ill.

Some participants also spoke of AN being useful in their friendship, like a Social Comfort Blanket. Nina explained that her AN made her feel more confident in social situations: “The time I needed to be…worrying about, what did they think of me, was consumed by my illness; “I put myself in more of those situations…in groups, I did get out more because I wasn’t as scared, because I had other [AN] thoughts on my mind”. Others also mentioned how recovering from AN was making it harder to socialise. Participants spoke about AN coming to define them and said that their illness gave them more confidence: “it made me a lot more confident when I first went back to uni…I felt I could make more friends” (Freya) Others talked about using AN as an excuse when feeling anxious about socialising.

Forming friendships with other individuals who also suffer from AN was discussed: “It’s good to be able to speak to people who are kind of like, have the same emotions or the same kind of thoughts as you” (Freya). Tess, however, spoke about feeling negative towards other patients. Several participants spoke about how being ill has helped them recognise the importance of their friendships or provided motivation to recover: “my family and friends are the people who support me and you realise that they’re the people that know you, and that value you” (Tanya).

Finally, participants spoke about their sense of self and self-confidence, and how this has changed since developing AN: “I kind of want to like find out who I am or create a person to be and then find friends, not find friends, but I mean discover them” (Olivia); “my ED kind of came to define me” (Teegan).

#### Social connectedness

Social Connectedness was the most prevalent theme found within the data, encompassing closeness of friendships, friendship difficulties, the process of making friends, friendship preferences and the positive qualities identified in participant’s friends. Friendship difficulties appeared to be particularly important to participants including specific conflict: “it was quite cliquey, one day you were in and one day you were out” (Danielle); other participants spoke of feeling indifferent about friendships or feeling detached from their friends: “I used to float between; I mean I didn’t really get along with anyone” (Emma).

Eight participants described having friendships which were positive in some way, for example having a best friend: “I’ve always got, like a best friend” (Tanya), close or long-standing friendships: “I would talk to them about everything and we were always together” (Teegan). Two participants (Nina and Olivia) described feeling lonely or isolated without their friends. All participant spoke about positive qualities of their friends: “I love my friends; I have such a good time with them and they mean a lot to me” (Danielle), which seemed particularly salient, given that a lot of the conversation was often focused on difficulties or negative experiences.

The sub-theme of dependency was present in half of the interviews: “I’ve become dependent on other people, it’s like I can’t be alone” (Danielle). Several participants describe having mixed feelings about their friendships, which seemed particularly important during analysis: It’s not that I don’t value them…but I can also go for weeks without talking to anyone” (Emma).

Participants expressed preferences for relationships beyond their immediate peer group. Two participants (Sam and Tess) indicated a preference for male friends: “I was friends with boys, who I got on well with”. Tess and Olivia mentioned being friends with animals while Emma preferred friendships with adults; “I didn’t like the kids that were there because I thought they were not fun…I find it easier to talk to them [adults], I can have better conversations, and they are more serious, not so petty”.

All participants spoke about making friends, either in terms of initiating friendships or the number of friends they had. Tanya found making friends easy, while others found it difficult to initiate friendship, which appeared to be a strong theme: “I didn’t like that whole getting to know someone period” (Emma); While some participants described having several friends, others had only a few.

#### Inflexibility

Inflexibility was identified in eight of the interviews, incorporating the subthemes of intolerance of uncertainty, need for control, perfectionism and difficulty with transitions. This seemed to be a relatively important theme, as many of the issues discussed impacted on social functioning. Uncertainty manifested as a need for structure in friendships: “other people were kind of, would be quite spontaneous, and I guess…I just wasn’t, I didn’t like things that weren’t planned” (Darcy) and anxiety in not knowing how long friendships would last. Participants described needing to take the lead in social situations and wanting social situations to be predictable: “I want to be in control of where I want to go. I want to know what is in the place” (Tanya).

Some participants described having an issue with perfectionism or a need to be productive: “we were all quite studious, like being very studious and wanting to do really well at school”. Participants spoke about difficulties transitioning from one school to another and the impact this had on their friendships: “It would be very tough and I wouldn’t want my mum to leave at all” (Tanya). Several participants spoke about losing friends when moving to secondary school and finding it hard to make new friends: “even though I left primary school with some of the friends that I had, they seemed to kind of…make more friends and there was a bigger group and I found that really overwhelming” (Darcy); “I found it quite difficult in the first couple of years to, like, establish a friendship group” (Freya).

#### Preferred social activity

Preferred Social Activity incorporated communication, interests and types of activity. Communication centred on different preferences for communicating with friends. Two participants discussed not liking phone calls: “I hate long phone calls, I like to keep them short” (Teegan). Several others indicated a preference for some form of electronic communication: “I’ll phone them or write to them, I don’t really seem many of them…I just try to make it really short” (Darcy).

Eight participants spoke about having shared interests with their friends, particularly when asked why they were friends with someone: “she was really into her beauty as well [as me]” (Tanya). Four participants, however, spoke about not being able to relate to others: “they were into thing that I wasn’t really into” (Nina); “I don’t have a lot of the experiences they’ve had” (Olivia). Sam spoke about needing time out from social groups due to finding them intense: “I needed time to come down from it”. All participants spoke about the types of activity they engaged in with their friends but for some, this centred on activities that required minimal social interaction: “I’d ride with some of my friends at the horses” (Tess). Three participants mentioned that they did not socialise outside of school when they were younger: “I didn’t really go out after school until about the third year [of secondary school]” (Sam) while others spoke of regular social contact with their friends.

## Discussion

The aim of this qualitative study was to explore the friendship experiences of females with AN, both before and after the onset of their illness. Five overarching themes were identified through analysis: Social Connectedness; Social Concern; Impact of AN; Preferred Social Activity and Inflexibility. All participants described difficulties with friendship prior to the onset of their ED and AN impacting on the quality of their friendships. The accounts given by participants support the cognitive-interpersonal maintenance model of AN [2] which proposes that difficulties with social interaction both predispose individuals to, and perpetuate the disorder. The findings are also in line with difficulties reported by younger patients [23], suggesting similarities in terms of social difficulties in both adults and younger people with EDs.

Social concern, the strongest theme identified, incorporated anxiety; fitting in; judgement and social exclusion, with participants describing social concern before the onset of their AN. The high correlation between anxiety disorders and EDs is well documented [27] and anxiety about social situations, in which the individual is exposed to possible scrutiny by others is a defining feature of social anxiety disorder [1]. Fear of negative evaluation accounts for a significant portion of the relationship between social anxiety and eating pathology [28]. Additionally, social appearance anxiety is a key risk factor for the development of ED symptoms [29]. While examining risk-factors was outside of the scope of the current study, qualitative accounts of some participants did allude to anxiety about appearance, with individuals describing feeling judged by others and worrying about how they looked. Social exclusion was also apparent, in line with previous findings which suggest that bullying increases the risk of developing AN [7].

Social Connectedness, including conflict in friendships relates to previous research, which associated conflict with the development of an ED [30]. Regarding making friends, our qualitative findings suggest that individuals with AN may find it hard to initiate friendships and may have smaller social networks, consistent with previous literature [4,5,13,17]. Several participants described finding it hard to make friends or having only a few friends as well as feeling lonely or disconnected from their peers, even prior to the onset of their illness. This suggests that the quality of friendships may be lower in individuals who develop AN, in line with previous research, which associated low-quality friendship with disordered eating [25]. Sharpe et al. [25] highlight the need for longitudinal studies to determine whether poor quality friendship precedes the development of ED pathology. Although the current study is not sufficient to determine whether difficulties with friendship do precede AN, insights from the participants interviewed appear to allude to this.

Difficulties with making and maintaining friends may have important clinical implications. Loneliness and negative interpersonal relationships may exacerbate ED symptoms [31] and thus hospitalisation may temporarily reduce social isolation by creating an interactive environment [18]. Individuals with AN report wanting meaningful personal relationships but struggle being in social situations [21]. The importance of aspects of life outside of work or study have been cited as meaningful motivators of recovery [22], therefore, patients can be encouraged to maintain contact with friends outside of hospital, particularly if they acknowledge positive aspects of friendships. Treatment should consider the inclusion of the wider social network to ensure that when leaving hospital, individuals are able to form and maintain meaningful relationships outside of the family network.

The impact of AN on friendships was particularly significant, given the possible treatment implications associated with this theme. The finding that all participants’ friendships have been impacted by the development of AN supports previous findings that individuals with AN have poor functioning and quality of life in the domain of social relationships [3,32–34]. The subtheme of AN as a Social Comfort Blanket is a concerning but important finding and is in line with a qualitative study which revealed that patients expressed beliefs that their AN made them feel protected and looked after [35]. This highlights the need for pro-anorectic beliefs to be addressed within treatment.

The theme of Preferred Social Activity was coined to describe communication preferences, interests and type of activity. In Doris et al. [17] study, participants showed a preference for electronic, as opposed to face-to-face communication, which was also the case in the current study. Doris et al. [17] suggest that difficulties with emotion recognition [36,37], evoked facial expression [36,38,39] and mirroring other people’s facial expression [40,41] may cause individuals with AN to avoid face-to-face communication. This may be due to difficulties with open-ended communication, highlighting a preference for more structured activity. Preference of electronic communication could be considered within inpatient treatment when making decisions about use of mobile phones and internet access. Patients who struggle with face-to-face contact may also benefit from additional support around visiting hours and planning for periods of leave and further research examining these provisions would be beneficial.

The themes identified in our study suggest some similarities with the ASD, in line with a previous study [17]. Females with ASD have been found to have difficulties maintaining friendships [42] and young people with ASD have fewer friends and engage in more solitary activities than their typically developing peers [43,44]. Children with ASD rate their friendships as poorer in quality than their peers but the level of satisfaction with friendship does vary [42]. Compared to their male counterparts, females with ASD may appear less-impaired to their teachers and are more able to maintain reciprocal social-interaction and integrate verbal and non-verbal communication [45]. Females with ASD may mimic their peers in order to camouflage some of their social difficulties and fit in with others [46]. The relationship between AN and ASD remains unclear, with suggestion that the ill state of AN may exacerbate or cause ASD-like traits [47] such as difficulties with social interaction and thus any conclusion regarding a specific relationship between AN and ASD cannot be drawn.

The need for structure was also present in the final theme of Inflexibility. Subthemes of intolerance of uncertainty and need for control could result from difficulties in the domains of Set Shifting and Central Coherence, established areas of difficulty in AN [48–51]. This inflexible and detailed thinking style is addressed within Cognitive Remediation Therapy [52] and emotional difficulties in Cognitive Remediation and Emotion Skills Training [53] and thus the current study highlights the importance of these interventions, particularly in relating neuropsychological style to everyday situations. Difficulties with transition supports existing literature which suggests that transitioning to college may represent a particular challenge for girls who already have disordered eating attitudes or behaviours [54–56]. The relationship between perfectionism and AN is also well-established [57,58] and novel research has begun to develop psychological interventions to target perfectionism in AN [59–62].

The findings of the current study should be considered in the context of certain limitations including the small sample size; while this is not uncommon in qualitative research, the findings cannot be generalised and may not reflect the views of other individuals with AN or other EDs, particularly individuals receiving outpatient treatment. Lack of generalisability may have also resulted from the use of purposive sampling as while effort was made to recruit a representative sample, this was based on the subjective judgement of the researcher. Although the interview specifically asked about experiences of friendship prior to the onset of AN, the data is based on retrospective reports which may not be entirely accurate, particularly as some participants had a protracted illness course. Future research would benefit from exploring friendship experiences in younger participants or in those at high risk of developing a clinical ED. While all individuals interviewed described difficulties with their friendship, this may not be unique to individuals with AN. Further studies would therefore benefit from comparing the experiences to HCs or individuals with other psychiatric disorders. In addition, previous research [25,30] has highlighted the role that depression may play in mediating the relationship between ED pathology and poor friendship quality. As depression was not controlled for in the current study, this cannot be commented on and this is a further limitation of the study’s methodology.

Several difficulties associated with friendship, identified from this analysis could be addressed within current treatments, for example, family-based treatment may benefit from considering the wider social system and the impact this may have on recovery [63]. Issues relating to isolation or loneliness may also be addressed with Occupational Therapy, or generally a practical approach, to actively increase the social activity that patients engage in. Similarly recovery-focused work, such as the Recovery Star [64,65], which highlights the role that relationships play in recovery may be beneficial for AN patients, although work with this population is in its infancy.

Finally, issues around others not understanding AN may be addressed through psychoeducation and carer’s work, which is widely used in EDs with good outcomes [66,67]. Cognitive behavioural approaches may be used to address social anxiety [68] or negative beliefs about body image or social comparison [69,70]. As mentioned, CRT, CREST and perfectionism based work may also be effective for targeting some of the difficulties described by the participants in this study. There are, however, still gaps in treatment provision, particularly to address friendship difficulties and social interaction. With the exception of a small pilot study [24], no specific interventions for AN have been developed to target friendship difficulties, which may be a viable treatment target.
